# Optimising the radiolabelling properties of technetium tricarbonyl and His-tagged proteins

**DOI:** 10.1186/2191-219X-4-14

**Published:** 2014-03-07

**Authors:** Adam Badar, Jennifer Williams, Rafael TM de Rosales, Richard Tavaré, Florian Kampmeier, Philip J Blower, Gregory ED Mullen

**Affiliations:** 1 Division of Imaging Sciences and Biomedical Engineering, King's College London, 4th Floor, Lambeth Wing, St Thomas’ Hospital, London SE1 7EH, UK; 2 Centre for Advanced Biomedical Imaging, Division of Medicine, University College London, London WC1E 6BT, UK; 3 Crump Institute for Molecular Imaging, Department of Molecular and Medical Pharmacology, David Geffen School of Medicine at the University of California–Los Angeles, Los Angeles, CA 90095-1735, USA

**Keywords:** Protein radiolabelling, Site-specific radiolabelling, 99mTc tricarbonyl, His-tag radiolabelling, Technetium-99 m

## Abstract

**Background:**

To date, the majority of protein-based radiopharmaceuticals have been radiolabelled using non-site-specific conjugation methods, with little or no control to ensure retained protein function post-labelling. The incorporation of a hexahistidine sequence (His-tag) in a recombinant protein can be used to site-specifically radiolabel with ^99m^Tc-tricarbonyl ([^99m^Tc(CO)_3_]^+^). This chemistry has been made accessible via a technetium tricarbonyl kit; however, reports of radiolabelling efficiencies and specific activities have varied greatly from one protein to another. Here, we aim to optimise the technetium tricarbonyl radiolabelling method to produce consistently >95% radiolabelling efficiencies with high specific activities suitable for *in vivo* imaging.

**Methods:**

Four different recombinant His-tagged proteins (recombinant complement receptor 2 (rCR2) and three single chain antibodies, α-CD33 scFv, α-VCAM-1 scFv and α-PSMA scFv), were used to study the effect of kit volume, ionic strength, pH and temperature on radiolabelling of four proteins.

**Results:**

We used 260 and 350 μL [^99m^Tc(CO)_3_]^+^ kits enabling us to radiolabel at higher [^99m^Tc(CO)_3_]^+^ and protein concentrations in a smaller volume and thus increase the rate at which maximum labelling efficiency and specific activity were reached. We also demonstrated that increasing the ionic strength of the reaction medium by increasing [Na+] from 0.25 to 0.63 M significantly increases the rate at which all four proteins reach a >95% labelling efficiency by at least fourfold, as compared to the conventional IsoLink® kit (Covidien, Petten, The Netherlands) and 0.25 M [Na+].

**Conclusion:**

We have found optimised kit and protein radiolabelling conditions suitable for the reproducible, fast, efficient radiolabelling of proteins without the need for post-labelling purification.

## Background

The specificity and affinity of recombinant proteins and antibodies targeted towards antigens make them highly attractive as a basis for radiopharmaceuticals for molecular imaging. To retain these key attributes, it is essential not to compromise the recognition function of the protein when radiolabelling. This can be achieved using site-specific labelling methods that exert maximum control over the number and site of modification(s) to the molecule, while still maintaining protein function. Such methodologies should give rise to homogeneous conjugates with reproducible chemical and pharmacological properties. They must ensure that the conjugation of the radiochelate is outside of the target binding site or at a distinct site known not to affect antigen binding. Ideally, radiolabelling would be achieved rapidly, under mild conditions, to a high specific activity and preferably in a simple one-pot kit-based method without the need for subsequent purification steps.

Waibel et al. [1] developed an elegant method that permits radiolabelling of proteins engineered with sequences of additional histidine residues known as His-tags. The His-tag was originally developed to facilitate purification of recombinant proteins using metal chelate-based affinity chromatography. Radiolabelling of His-tags could be achieved with technetium-99 m (^99m^Tc) in its stable + I oxidation state in the form of ^99m^Tc-tricarbonyl ([^99m^Tc(CO)_3_]^+^). This organometallic complex is produced in the form of its aqua ion [^99m^Tc(CO)_3_(H_2_O)_3_]^+^ in a one-step reaction by reduction of the generator-eluted form of ^99m^Tc, sodium pertechnetate ([^99m^TcO_4_]^-^). Histidine has been demonstrated to be the favoured [^99m^Tc(CO)_3_]^+^-binding ligand among amino acids and labelling efficiency and stability increase with increased number of engineered histidines [2]. Direct labelling of non-His-tag proteins with [^99m^Tc(CO)_3_]^+^ has previously resulted in poor stability and low labelling efficiency and specific activity, indicating that other potential amino acid side chains donor groups such as thiol, thioether, carboxylate and amine do not make a significant contribution in the absence of histidines [3-9]. Modification of the His-tag sequence from HHHHHH to HEHEHE in order to improve tracer biodistribution also resulted in reduced labelling efficiency [10,11]. In contrast, by engineering an additional cysteine seven amino acids downstream of the His-tag, Tavaré et al. demonstrated an improvement in labelling efficiency and specific activity compared to His-tag alone [12] and showed for the first time that the radiolabelling is indeed site-specific to the His-tagged region of the protein by carrying out tryptic digest and mass spectrometry on a His-tagged protein labelled with [Re(CO)_3_]^+^. The rhenium complex was only present in His-tag-containing fragments [12]. Furthermore, we recently demonstrated that the engineered cysteine that increases radiolabelling efficiency is also involved in the coordination of the rhenium tricarbonyl [13]. Several variants on labelling conditions have been studied to optimise the use of [^99m^Tc(CO)_3_]^+^-labelling of His-tagged proteins, and there is a general agreement in the literature that labelling at neutral pH, high protein concentration and high temperature increase the rate of radiolabelling (see Additional file 1: Table S1) [1,2,12,14-26]. These conditions, however, exclude proteins that are susceptible to aggregation or loss of function at high temperatures and concentrations or require particularly high specific activity [15,23,25]. To make this labelling chemistry readily accessible, a kit-based formulation has until recently been distributed by Mallinckrodt (subsidiary of Covidien, Petten, The Netherlands) under the trademarked name IsoLink. Since its introduction, IsoLink has supported the development of numerous protein-based imaging tracers. Despite the intent to provide a practical, simple and versatile radiolabelling method, there has been a large variation in labelling efficiencies reported in the literature, likely due to variations in protein/peptide properties and labelling conditions (see Additional file 1: Table S1), and no clinical trial has been performed to date with proteins labelled using the tricarbonyl labelling method.

The IsoLink kit comes in a 10-mL glass vial containing lyophilised reagents and is designed to reduce up to 3.7 GBq of [^99m^TcO_4_]^-^ in a final volume of 1 mL. With the current composition, to achieve maximum specific activity (crucial for *in vivo* imaging), the protein must be added to this total volume, leading to low protein concentration and hence inefficient labelling [12] and high wastage. Recombinant proteins used in R&D are often precious, produced in low yield and available in small quantities and may be difficult to concentrate due to aggregation, precipitation or loss on columns or membranes. Furthermore, for preclinical work, volumes above 200 μL are not desirable or feasible for injection into mice (one tenth of total blood volume). The current kit necessitates an avoidable and time-consuming protein concentration step or leads to low specific activity. Thus, while labelling via the [^99m^Tc(CO)_3_]^+^ method may be one of the most promising site-specific methods currently available for recombinant protein tracer development, the IsoLink kit in its current form is not optimal for routine use in preclinical research or for future clinical imaging. It needs to be optimised to achieve reproducible high labelling efficiencies (LE) and specific activities (SA) of recombinant proteins, without wasting large amounts of protein and ^99m^Tc.

In this paper, we address how the kit could be further optimised to increase specific activity and radiolabelling efficiency of His-tagged proteins, at suitable labelling rates with reduced wastage of protein and radioactivity. We used four different His-tagged proteins to study the effect of various radiolabelling conditions.

## Methods

### Materials

IsoLink kits (generously provided by Covidien, Petten, The Netherlands) consisted of lyophilised formulation in an N_2_-flushed 10-mL glass vial, containing 8.5 mg sodium tartrate Na_2_C_4_H_4_O_6_, 2.85 mg sodium tetraborate Na_2_B_4_O_7_, 7.15 mg sodium carbonate Na_2_CO_3_ and 4.5 mg sodium boranocarbonate Na_2_H_3_BCO_2_. Kits are now available from an alternate supplier (Centre of Radiopharmaceutical Research at the Paul Scherrer Institute in Switzerland) or can be prepared according to Waibel et al. [1].

His-tagged proteins used in this study were (1) recombinant complement receptor 2 (rCR2) (16 kDa) recombinant protein [27], (2) α-CD33 scFv (29 kDa) [28], (3) α-PSMA scFv (27.7 kDa) [29] and (4) α-VCAM-1 scFv (28.8 kDa) [30]. Protein concentration was measured by UV spectrometry using a Nanodrop device (ThermoScientific, Loughborough, Leicestershire, LE, UK) and a molar extinction coefficient of the respective protein determined by ProtParam [31].

### Preparation of [^99m^Tc(CO)_3_]^+^

Three methods were used and compared in the production of [^99m^Tc(CO)_3_]^+^: a standard method employing an IsoLink kit according to the manufacturer's instructions (method 1), a method using the IsoLink kit with a reduced volume of [^99m^TcO_4_]^-^ solution (method 2) and a new subdivided kit formulation (method 3).

#### *Methods 1 and 2*

Up to 2.5 GBq [^99m^TcO_4_]^-^ in 1 mL (method 1) or in 350 to 400 μL (method 2) of saline was added to the kit and heated for 30 min at 100°C. The vial was then allowed to cool to room temperature (RT) and the solution neutralised with 145 to 160 μL (methods 1 and 2) of 1 M HCl to pH approximately 7.5 giving a total volume of 1,145 to 1,160 μL (method 1) or 495 to 560 μL (method 2), respectively.

#### *Method 3*

IsoLink kits were reconstituted in 1 mL ultrapure milliQ water (Millipore, Billerica, MA, USA), degassed with nitrogen and subdivided into aliquots of 260 μL in microcentrifuge tubes with rubber ring-sealed screw caps (Nalgene, Rochester, NY, USA) under anaerobic environment. Aliquots were snap-frozen in liquid nitrogen, freeze-dried overnight, then stored at -80°C. Upon use, kits were allowed to thaw to RT, and up to 1 GBq [^99m^TcO_4_]^-^, generator elulate (100 μL) was added and heated for 30 min at 100°C. The microcentrifuge tube was then allowed to cool to RT and the solution neutralised with 25 μL of 1 M HCl to pH approximately 7.5 giving a total volume of 125 μL of [^99m^Tc(CO)_3_]^+^.

#### *Quality control*

To determine the radiochemical purity of the [^99m^Tc(CO)_3_]^+^ produced, glass-backed silica gel 60 F254 thin layer chromatography (TLC) plates (3 cm × 7.5 cm, Merck™, Darmstadt, Germany) were used with a mobile phase of 1% HCl in methanol. Plates were analysed with a gamma ray TLC scanner (Lablogic, South Yorkshire, UK). Purity was expressed as a ratio of the [^99m^Tc(CO)_3_]^+^ peak integral (*R*_f_ = 0.2 - 0.8) to integral of the whole chromatogram (including *R*_f_ = 0 for ^99m^Tc colloids and *R*_f_ = 0.9 for unreduced [^99m^TcO_4_]^-^).

### [^99m^Tc(CO)_3_]^+^ radiolabelling of His-tagged recombinant proteins

To 100 μL of rCR2 (1 mg/mL, 62.5 μM) or α-CD33 scFv (1 mg/mL, 34.5 μM), or 20 μL of α-PSMA scFv (1 mg/mL, 36.1 μM) or α-VCAM-1 scFv (1 mg/mL, 34.7 μM) in PBS (0.14 M [Na^+^]), a 20-μL solution of [^99m^Tc(CO)_3_]^+^ (prepared according to method 2 above for CD33 scFv, PSMA scFv and VCAM-1 scFv and method 3 for rCR2) was added. Each protein was radiolabelled in a final solution containing low, medium and high concentrations of Na^+^: 0.25 M [Na^+^] (low), 0.44 M [Na^+^] (medium) and 0.63 M [Na^+^] (high). The Na^+^ concentration of the [^99m^Tc(CO)_3_]^+^ solution was adjusted with NaCl solution before being added to the protein. The concentration and volume of NaCl solution used was calculated according to the concentration of the Na^+^ required in the final protein-[^99m^Tc(CO)_3_]^+^ solution after addition of [^99m^Tc(CO)_3_]^+^ (see Additional file 1 for [Na^+^] calculations). Final protein concentrations were 0.85 mg/mL rCR2 (53.1 μM), 0.42 mg/mL α-CD33 scFv (14.5 μM) and α-PSMA scFv (15.2 μM), and 0.68 mg/mL α-VCAM-1 scFv (23.6 μM). The labelling reaction was carried out at 37°C and monitored for 2 h with labelling efficiency determined at 5, 15, 30, 60, 90, and 120 min using iTLC-SA paper with citrate buffer as the mobile phase. This system provided a definitive separation between the [^99m^Tc(CO)_3_]^+^-labelled peptide (*R*_f_ = 0) and unbound [^99m^Tc(CO)_3_]^+^ and unreduced ^99m^TcO_4_^-^ (both with *R*_f_ = 1). The large separation between the peaks enabled the iTLC-SA strips to be cut in half and both the solvent front and baseline segments analysed using a gamma counter. Radiolabelling efficiency was expressed as a ratio of the [^99m^Tc(CO)_3_]^+^ protein-labelled peak integral (*R*_f_ = 0) to the integral of the whole strip including unbound [^99m^Tc(CO)_3_]^+^ and unconverted [^99m^TcO_4_]^-^ (*R*_f_ = 1). The influence of pH, protein concentration and reaction temperature was evaluated using rCR2 and can be found in Additional file 1.

### Determining site specificity with a His-tag challenge

A non-His-tagged protein (C2Ac [12]) was allowed to compete for radiolabel with a His-tagged protein (α-CD33 scFv) by incubating an equimolar mixture of both proteins (55 μM:55 μM) in a one-pot radiolabelling reaction using the IsoLink kit method 2 described above. As controls, the proteins were radiolabelled separately using 55 μM concentrations. The reactions were allowed to incubate for 1 h at 37°C, after which 5 μL samples were taken from each reaction mixture and loaded onto an SDS-PAGE gel (12% NuPage gel, Invitrogen, Paisley, PA, UK). After the gel was run, a reference lane with molecular weight markers was spotted with radioactivity at the 15- and 30-kDa bands. After protein separation via electrophoresis, phosphor screens (Perkin Elmer, Shelton, CT, USA) were exposed for 10 s, followed by imaging using a Cyclone® phosphorimager system (Perkin Elmer). The gel was analysed using ImageJ software (NIH, Bethesda, MD, USA), and the fraction of total radioactivity bound to each of C2Ac and α-CD33 scFv was determined via densitometry of the protein bands.

## Results

### Reducing the volume of the IsoLink kit increases the [^99m^Tc(CO)_3_]^+^ concentration while maintaining its radiochemical purity

**Figure 1:**
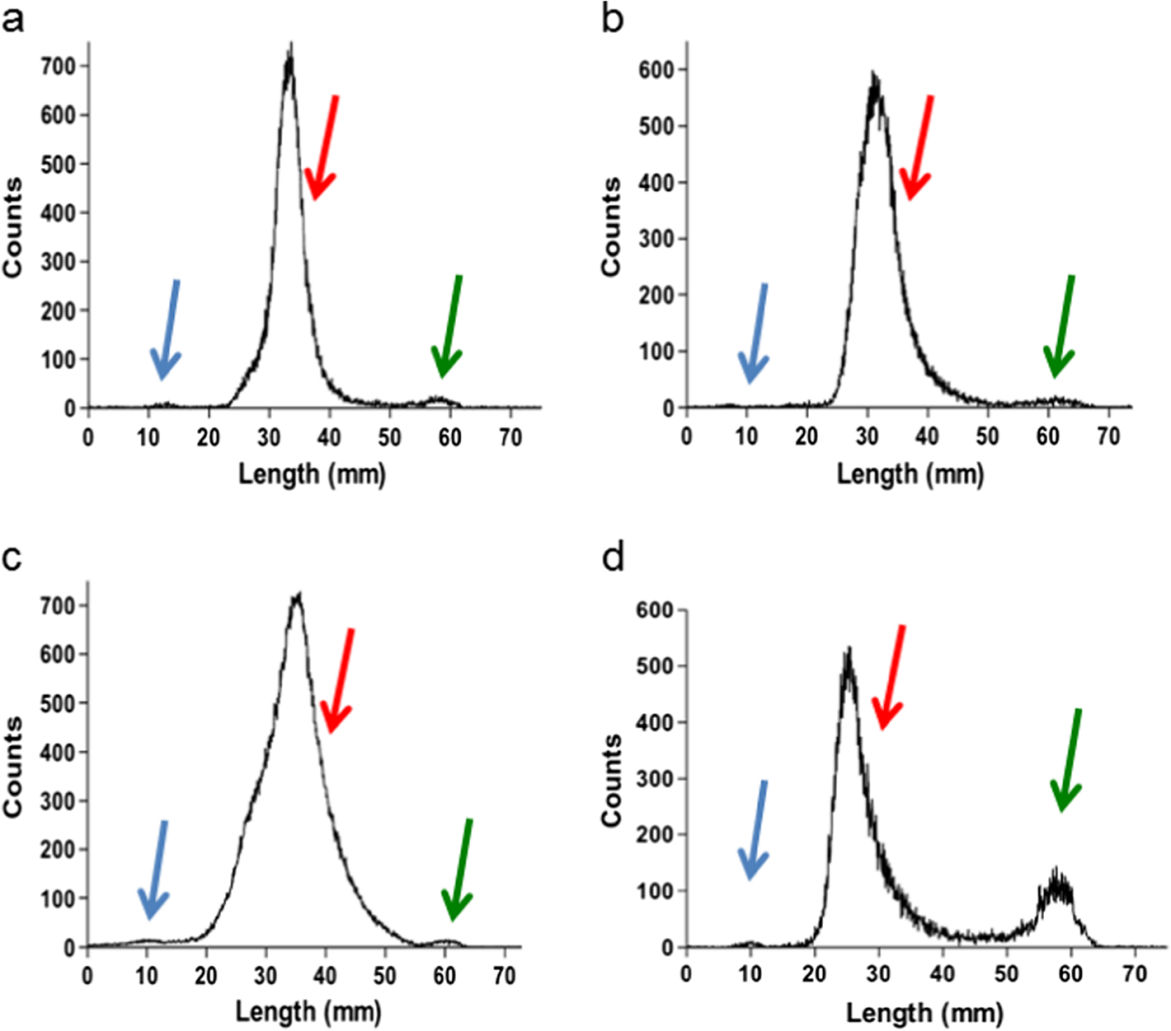
**TLC quality control of [**^**99m**^**TcO**_**4**_**]**^**- **^**reduction to [**^**99m**^**Tc(CO)**_**3**_**]**^**+**^**.***R*_f_ = 0.9 for [^99m^TcO_4_]^-^ (green arrow), *R*_f_ = 0 for ^99m^Tc colloids (blue arrow) and *R*_f_ = 0.2 to 0.8 for [^99m^Tc(CO)_3_]^+^ (red arrow). **(a)** Commercial IsoLink kit with 1 mL [^99m^TcO_4_]^-^ (method 1), **(b)** modified IsoLink kit with 350 μL [^99m^TcO_4_]^-^ (method 2) and **(c)** adapted kit with 100 μL [^99m^TcO_4_]^-^ (method 3). **(d)** Commercial IsoLink kit dissolved with 260 μL [^99m^TcO_4_]^-^. Kits **(a, b, c)** result in >98% radiochemical yield for [^99m^Tc(CO)_3_]^+^ formation, whereas kit **(d)** resulted in 82% ± 2.9% radiochemical yield for [^99m^Tc(CO)_3_]^+^ formation.

Labelling at high protein and [^99m^Tc(CO)_3_]^+^ concentration is essential to achieve maximum SA and LE within a suitable timeframe (see Additional file 1: Figure S1) and to avoid the need for post-labelling purification. In method 2, we reduced the volume of the IsoLink kit from the standard 1 mL (method 1 above) to 350 to 400 μL by adding a more concentrated solution of [^99m^TcO_4_]^-^. The radioactivity concentration in typical clinical generator eluates is easily sufficient to provide the required activity for clinical scanning in this reduced volume. We established that a minimum of 350 μL was required to completely dissolve the kit reagents. Reconstituting the standard IsoLink kit with a volume <350 μL while heating to 100°C led to precipitation and yielded only 82% ± 2.7% conversion of [^99m^TcO_4_]^-^ to [^99m^Tc(CO)_3_]^+^ (Figure 1d), whereas reconstituting with 350 μL or more gave no precipitate, a radiochemical yield of >97% ± 1.6% (Figure 1a) and a radioactivity concentration of approximately 5 to 6 GBq/mL after neutralisation of the [^99m^Tc(CO)_3_]^+^ solution to pH 7.4 with 1 M HCl (method 1 resulted in a radioactivity concentration of 2 to 3 MBq/μL). In method 3, we further reduced the kit volume by subdividing the kit into 260-μL freeze-dried aliquots, each able to reduce up to 1 GBq [^99m^TcO_4_]^-^ in 100 μL resulting in a radioactivity concentration of approximately 8 GBq/mL after neutralisation. Quality control was carried out comparing the radiochemical purity of [^99m^Tc(CO)_3_]^+^ in the three kit formulations used. Using TLC, it was demonstrated that yield and purity of [^99m^Tc(CO)_3_]^+^ was the same (>97%) using methods 1, 2 and 3 (Figure 1a, b, c).

### High [Na^+^] improves specific activity and labelling efficiency when radiolabelling His-tagged proteins with [^99m^Tc(CO)_3_]^+^

**Figure 2:**
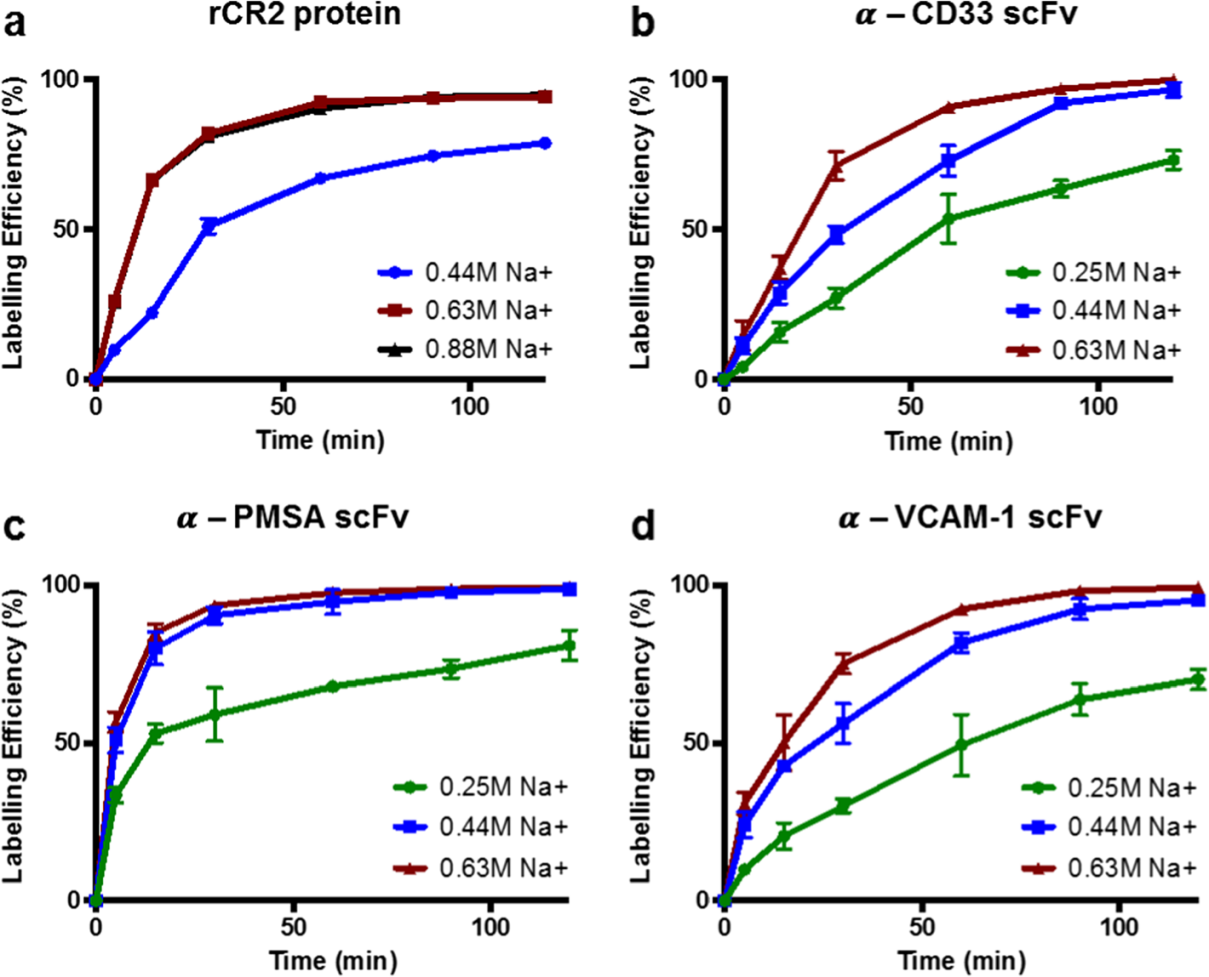
**Effect of [Na**^**+**^**] on [**^**99m**^**Tc(CO)**_**3**_**]**^**+ **^**radiolabelling of His-tagged proteins.** Labelling efficiencies (*n* = 3) of **(a)** rCR2, **(b)** α-CD33 scFv, **(c)** α-PSMA scFv and **(d)** α-VCAM-1 scFv proteins were determined at 5, 15, 30, 60, 90 and 120 min and represented as a percentage of incorporated [^99m^Tc(CO)_3_]^+^. Error bars represent the standard deviation of the mean of the triplicate. Where error bars are not visible, they are smaller than the data points.

To evaluate the influence of [Na^+^] on the LE of His-tagged proteins with [^99m^Tc(CO)_3_]^+^, four His-tagged proteins were radiolabelled at controlled [Na^+^] ranging from 0.25 to 0.63 M (with 0.44 M being the [Na^+^] in 1 mL of 0.9% saline [^99m^TcO_4_]^-^) using [^99m^Tc(CO)_3_]^+^ preparation method 3 above as a basis. We demonstrated that at given incubation times and temperatures, the total [Na^+^] affects SA and LE of the recombinant proteins when radiolabelling with [^99m^Tc(CO)_3_]^+^ (Figure 2, Tables 1 and 2). To further clarify the correlation between [Na^+^] and improved LE, the comparison was made between the time taken to reach a 50% radiochemical yield for the proteins at the different [Na^+^]. For the α-CD33 scFv, α-PSMA scFv and α-VCAM-1 scFv proteins, an increase in [Na^+^] from 0.25 to 0.63 M decreases the time required to reach a 50% radiochemical yield by at least 66% (Table 1). The positive correlation between LE and SA and the [Na^+^] in the radiolabelling solution is evident. Furthermore, for the *in vivo* application of [^99m^Tc(CO)_3_]^+^:protein bioconjugates, a 95% radiochemical purity is typically accepted as a threshold for clinical acceptability. Often a purification step is required in the process of producing the radiolabelled proteins (see Additional file 1: Table S1) to achieve this threshold. Table 2 demonstrates that increasing the [Na^+^] to 0.63 M enables the 95% radiochemical yield threshold to be reached within a shorter time (maximum 75 min for the proteins used here), after which a purification step is not required prior to its *in vivo* use. At low salt (0.25 M), a radiochemical yield of 95% is not achievable after 2 h of incubation with [^99m^Tc(CO)_3_]^+^. The rCR2 protein was further analysed at an increased [Na^+^] of 0.88 M. However, in this instance, there was no further positive impact on the LE (data not shown).

**Table 1:** Time taken to reach 50% LE of four His-tagged proteins at varying [Na+]

**His-tagged protein**	**0.25 M [Na+]**	**0.44 M [Na+]**	**0.63 M [Na+]**
rCR2	-	34 ± 1.4 min	10.2 ± 0.1 min
α-CD33 scFv	58.5 ± 6.3 min	31.7 ± 3.5 min	19.5 ± 2.4 min
α-PMSA scFv	15.2 ± 3.8 min	4.7 ± 0.8 min	3.9 ± 0.5 min
α-VCAM-1 scFv	65.5 ± 10.5 min	19.9 ± 0.2 min	13.2 ± 2.6 min

**Table 2:** Time taken to reach 95% LE of four His-tagged proteins at varying [Na+]

**His-tagged protein**	**0.25 M [Na+]**	**0.44 M [Na+]**	**0.63 M [Na+]**
rCR2	-	N/A	71.1 ± 3.8 min
α-CD33 scFv	N/A^a^	106.8 ± 2 min	68.9 ± 1.2 min
α-PMSA scFv	N/A^b^	51.5 ± 14 min	34.6 ± 6.4 min
α-VCAM-1 scFv	N/A^c^	110.8 ± 5.1 min	73.2 ± 3.8 min

^a^LE for α-CD33 scFv at 75 min was approximately 51.3%; ^b^LE for α-PMSA scFv at 75 min was approximately 65.8%; ^c^LE for α-VCAM-1 scFv at 75 min was approximately 52.7%.

### Non-specific binding of [^99m^Tc(CO)_3_]^+^ does not occur in the presence of a His-tag

**Figure 3:**
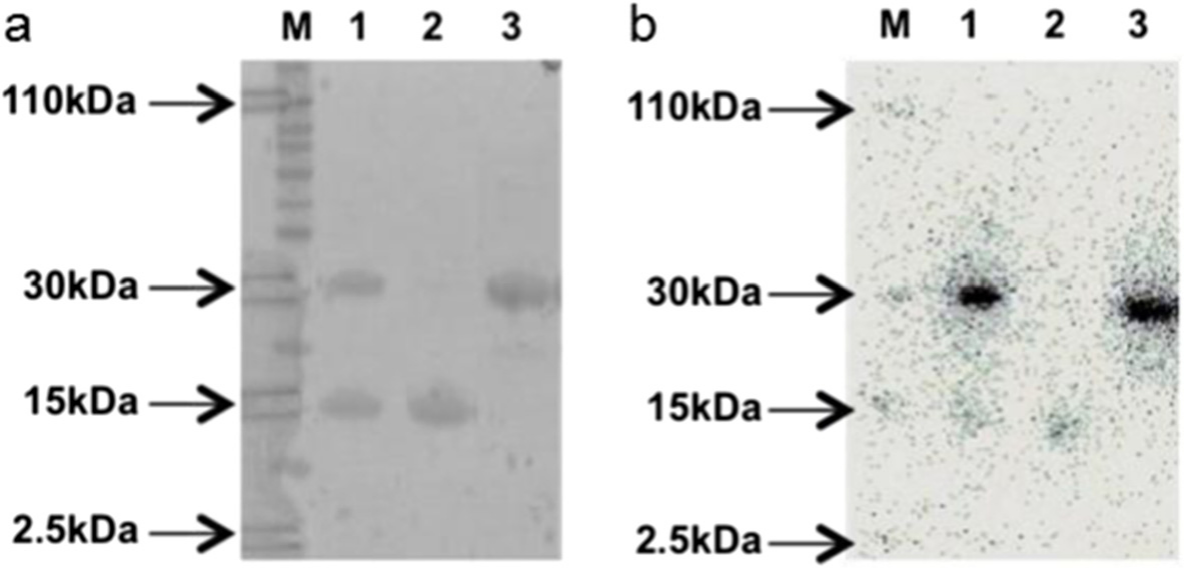
**Specificity of [**^**99m**^**Tc(CO)**_**3**_**]**^**+ **^**to His-tagged protein was demonstrated via (a) SDS-PAGE followed by (b) phosphor imaging.** Non-His-tagged protein (C2Ac, 15 kDa) was co-incubated with His-tagged protein (α-CD33 scFv, 29 kDa) and [^99m^Tc(CO)_3_]^+^. The proteins were then separated using SDS-PAGE, and radiolabelling was visualised using phosphor imaging. Lanes M, molecular weight marker; lanes 1, C2Ac + α-CD33 scFv; lanes 2, C2Ac; lanes 3, α-CD33 scFv. Radioactive markers, indicated by black arrows, were placed at 2.5, 15, 30 and 110 kDa.

We and others have previously shown that [^99m^Tc(CO)_3_]^+^ can bind non-specifically with a low labelling efficiency (approximately 10%) to proteins in the absence of a His-tag [12,2]. In this study, a competitive binding assay was developed by co-incubating equimolar amounts of two proteins, one His-tagged and the other non-His-tagged, with [^99m^Tc(CO)_3_]^+^. The radiolabel preferentially bound to the His-tagged protein by a factor of at least 50. Densitometry of the phosphor image indicated >98% ± 1.8% of [^99m^Tc(CO)_3_]^+^ was bound to the His-tagged protein (α-CD33 scFv) after 1 h incubation, whereas <2% ± 0.9% was complexed ‘non-specifically’ to the non-His-tagged protein C2Ac (Figure 3).

## Discussion

Technetium-99 m is well suited for small (<55 kDa) protein-based tracers (glomerular filtration molecular weight cutoff approximately 45 kDa below which the tracer has a markedly reduced blood clearance half-life). Its 6-h half-life is sufficiently long to carry out radiolabelling and allow for slower blood clearance of minutes to hours as compared to small molecules with seconds to minutes. Furthermore, ^99m^Tc is readily available at low cost from transportable ^99^Mo/^99m^Tc generators and has favourable photon energy for imaging while minimising patient radiation-absorbed dose. To radiolabel proteins with ^99m^Tc, the tricarbonyl method developed by Waibel et al. [1] offers a convenient solution enabling labelling without prior modification with bifunctional chelators, under mild conditions in as little as 60 min, and has recently supported the development of new radiopharmaceuticals [12,27].

A number of studies have demonstrated good stability of [^99m^Tc(CO)_3_]^+^:His-tagged protein complex, with minimal transchelation or loss of bound activity, by challenging with high concentrations of histidine and cysteine or incubating in serum [12,14,16,17]. Our group has recently shown that the complex specifically binds to the His-tag, demonstrated by the fact that rhenium tricarbonyl labelled His-tag protein post-tryptic digest is only bound to peptide fragments containing a His-tag [12]. Previous reports have indicated that non-His-tag proteins can be radiolabelled with [^99m^Tc(CO)_3_]^+^[4] albeit with relatively low efficiency, but little or no challenge or stability data was reported. By using a competitive binding assay, here we demonstrated that the technetium tricarbonyl indeed binds selectively to a His-tagged protein in the presence of a non-His-tagged protein and little or no (<2% ± 0.9%) non-specific labelling was observed.

Low radiolabelling efficiency (<95%) of radiopharmaceutical is problematic, with any unlabelled material competing for target, hampering quantitative measurements and imaging sensitivity, and requiring an additional purification step prior to imaging. High SA enables a radioactive signal using a low concentration of radiopharmaceutical well below the pharmacologically active dose and is crucial for imaging low-concentration targets. Radiolabelling the recombinant proteins according to the IsoLink instructions resulted in relatively poor SA and LE due to excessive dilution of the protein (Additional file 1: Figure S1).

As previously reported, we found that protein concentration, pH and temperature play a key role in achieving maximum SA and LE (Additional file 1: Figure S1) when labelling with technetium tricarbonyl. Achieving the optimal conditions however is currently hampered with the commercial IsoLink kit. To overcome this, we prepared modified kits that can produce a [^99m^Tc(CO)_3_]^+^ solution with radiochemical purity equal to that of the unmodified kits but with higher radioactivity concentration. The lower volume means that the radiolabelled product can conveniently be used for preclinical *in vivo* studies, making more economical use of both protein and radioactivity. The higher protein and radionuclide concentration leads to a faster labelling reaction, in turn to achieving higher SA and LE in the same incubation time, or a shorter incubation time to reach the same SA and LE. Importantly, it reduces the incubation time, temperature and amount of protein needed to reach 95% LE (the threshold at which radiochemical purity is typically regarded as adequate for *in vivo* use) and hence makes it easier to avoid the need for post-labelling purification steps which can waste time and cause losses due to irreversible binding to columns and filters.

We also found that total [Na^+^] in the reaction mixture affects SA and LE, and this effect can be exploited by increasing [Na^+^] to increase the rate at which maximum SA and 95% LE are reached by up to fourfold. IsoLink kits contain a significant amount of Na^+^, 0.31 mmol, and the [Na^+^] of the final protein: [^99m^Tc(CO)_3_]^+^ reaction mixture is dependent on the volume of the [^99m^Tc(CO)_3_]^+^ solution added (see Additional file 1 for [Na^+^] calculations).

Using the conventional IsoLink kit, rates of labelling reactions and times to reach maximum SA and LE vary between proteins. These variations may be due to His-tag accessibility in the protein's tertiary structure or a charge effect from adjacent amino acids interacting with the positively charged tricarbonyl core. Increasing the ionic strength or [Na^+^] in the reaction mixture may contribute to a kinetically favourable binding environment. For example, at pH 7.5, one would expect one or more of the histidines in the His-tag to be positively charged and if, as reported in the literature, the technetium tricarbonyl precursor is also positively charged, the increase in the ionic strength of the buffer may dissipate the ionic repulsion between the species thus lowering the activation energy of binding.

## Conclusions

In conclusion, the standard IsoLink kit, while reliably producing the technetium tricarbonyl precursor in high yield, is not optimal in scale for the routine and economic radiolabelling of different His-tag recombinant proteins at high specific activity for *in vivo* use. Each protein will need optimisation to achieve optimal SA and LE, and in some cases, adequate SA and LE may not be reached for routine clinical or preclinical use and purification steps are then required. By introducing modifications to the kit, it is possible to achieve reproducible and robust radiolabelling with optimal SA and LE in shorter incubation times and with smaller quantities of protein, avoid post-labelling purification steps and make the most efficient use of both protein and ^99m^Tc, improving the prospects for clinical translation and commercialisation of the tricarbonyl kit.

## Abbreviations

LE: labelling efficiency; SA: specific activity.

## Competing interests

The authors declare that they have no competing interests.

## Authors’ contributions

AB and JW contributed equally to this work and are joint first authors. AB carried out the protein radiolabelling, kit formulation, challenge assays, and drafted the manuscript. JW carried out protein radiolabelling, kit formulation, and drafted the manuscript. RR contributed to kit formulation and HPLC and TLC quality control. RT and FK contributed to production and purification of proteins. PB and GM conceived of the study, participated in the design of the study and helped to draft the manuscript. All authors read and approved the final manuscript.

## Supplementary Material

Additional file 1 **Supporting information.** Here, we provide a tabulated literature review summarising labelling conditions and outcomes of His-tagged proteins radiolabelled with ^99m^Tc-tricarbonyl (Additional file 1: Table S1). Additionally, we describe the effect of varying conditions (pH, temperature and protein concentration) when labelling a His-tagged protein with the IsoLink kit (Additional file 1: Figure S1). Finally, the calculation steps we used to calculate the [Na^+^] throughout this paper are outlined. Click here for file
