# Supplementary material for: Optimising the radiolabelling properties of technetium tricarbonyl and His-tagged proteins
Source: EJNMMI Res. 2014 Mar 7;4:14. doi: 10.1186/2191-219X-4-14 (PMC4015829; doi:10.1186/2191-219X-4-14)
Supplement: Additional file 1 — Supporting information. Here, we provide a tabulated literature review summarising labelling conditions and outcomes of His-tagged proteins radiolabelled with 99mTc-tricarbonyl (Additional file 1: Table S1). Additionally, we describe the effect of varying conditions (pH, temperature and protein concentration) when labelling a His-tagged protein with the IsoLink kit (Additional file 1: Figure S1). Finally, the calculation steps we used to calculate the [Na+] throughout this paper are outlined. [file 2191-219X-4-14-S1.docx]

**SUPPORTING INFORMATION (additional file1)**

**Optimising the radiolabelling properties of technetium tricarbonyl and His-tagged proteins**

Adam Badar^†, ‡^, Jennifer Williams^†^, Rafael Torres^†^, Richard Tavaré^†,§^, Florian Kampmeier^†^, Philip Blower^†^, Gregory E. D. Mullen^†,*^

^†^ Division of Imaging Sciences, Kings College London, St Thomas’ Hospital; ^‡^ Current address: Centre for Advanced Biomedical Imaging, University College London; ^§^ Current address: Crump Institute for Molecular Imaging, Department of Molecular and Medical Pharmacology, UCLA, Los Angeles, CA, USA; * Dr. Gregory Mullen, Division of Imaging Sciences, King’s College London, 4^th^ Floor Lambeth Wing, St Thomas’ Hospital, London SE17EH, greg.mullen@kcl.ac.uk, +44(0)20771888371; Author contributions: Adam Badar and Jennifer Williams contributed equally.

**Table of Contents**

Tabulated literature review summarising labelling conditions and outcomes of His-tagged proteins radiolabelled with ^99m^Tc-tricarbonyl…………………………………………...**P3-4**

Effect of protein concentration, pH, and temperature on [^99m^Tc(CO)_3_]^+^ radiolabelling of a His-tagged protein…………………………………………………………………………**P5-6**

Calculation of total Na^+^ concentration when radiolabelling His-tagged proteins with [^99m^Tc(CO)_3_]^+^ ……………………………………………...…………………….………**P7-8**

Table S1 is a tabulated literature review summarising labelling conditions and outcomes of His-tagged proteins radiolabelled with ^99m^Tc-tricarbonyl.

**Table S1: tubulated literature review summarising labelling conditions and outcomes of His-tagged proteins radiolabelled with ^99m^Tc-tricarbonyl**

| **His-tagged protein** | **[^99m^Tc(CO)3]+ preparation** | **Protein labelling conditions** | **LE** | **Purification** | **RCP** | **SA (MBq/µg)** | **SA (MBq/µL)** | **Ref.** |
| --- | --- | --- | --- | --- | --- | --- | --- | --- |
| His-CNA35 | Isolink^a^ -(1mL; <2.6GBq) | Activity added: <2.6GBq, final volume: >1.1mL [^99m^Tc(CO)_3_]^+^, [Final protein]: nd, reaction time: 1.5hr, temp: 37C, extra: blow dry rxn with N_2_ to reduce volume | 40-50% | Yes | 95-99% | 8.9-10.4 | >0.25 | 14 |
| 15KDa nanobody | Isolink^a^ - (1mL; <3.7GBq) | Activity added: <740 MBq, final volume: 0.65 mL, [final protein]: 0.33mg/mL, reaction time: 1hr, temp: 52°C | 70-95% | Yes | >95% | nd^e^ | nd | 15 |
| Annexin V | Homemade kit^c^ (1mL; <740 MBq); [^99m^Tc(CO)_3_]^+^ RCP >90% | Activity added: <300 MBq, final volume: 0.75mL, [Final protein]: 0.67mg/mL, reaction time: 50min, temp: 50 °C, | >95% | nd | nd | 0.74 | 0.99 | 16 |
| *α-* HER2 affibody | Isolink^a^ - (0.6mL; nd MBq) | Activity added: nd, final volume: 0.08mL, [Final protein]: 0.6mg/mL, reaction time: 40min, temp: 50°C | 61% | Yes | >97% | nd | nd | 17 |
| Barnase:Barstar scFv | Homemade kit^b^ | Activity added: nd, final volume: 0.08mL, [Final protein]: 0.2mg/mL, reaction time: nd, temp: nd | >95% | nd | nd | 2 | 0.2 | 18 |
| Annexin V | Isolink^a^ - (1mL; 370MBq) | Activity added: 37MBq, final volume: 0.250mL, [Final protein]: 0.4mg/mL, reaction time: 1hr, temp: 37°C | <80% | Yes | >96% | 0.74 | 0.05 | 2 |
| C2AcH | Isolink^a^ - (1mL; <2.6 GBq); [^99m^Tc(CO)_3_]^+^ RCP >90% | Activity added: 700MBq, final volume: 0.2mL, [Final protein]: 0.5mg/mL, reaction time: 30min, temp: 37°C | >95% | Yes | 100% | 7 | 0.33 | 12 |
| DARPin | Isolink^a^ - (1mL) | Activity added: nd, final volume: nd, final protein Concentration: nd, reaction time: 1hr, temp: 37°C | nd | nd | nd | nd | 2.77 | 19 |
| L19 scFv | Homemade kit^d^ | Activity added: 37MBq, final volume: 0.331mL, [Final protein]: 0.3mg/mL, reaction time: 1hr, temp: 37°C | 47% | Yes | 97% | 0.26 | nd | 20 |
| *α-MCAM scFv* | Isolink^a^ - (1mL; 370MBq) | Activity added: 31MBq, final volume: 0.12mL, [Final protein]: 0.83mg/mL, reaction time: 1hr, temp: 37°C | nd | Yes | nd | 0.37 | 0.185 | 21 |
| M40 scFv | Isolink^a^ | Activity added: nd, final volume nd, [Final protein]: nd, reaction time: 1hr, temp: 37°C | <85% | Yes | >95% | 9.3 | nd | 22 |
| MFECP1 | Homemade kit^b^ (0.5mL < 200MBq), [^99m^Tc(CO)_3_]^+^ RCP 99% | Activity added: nd, final volume: nd, [Final protein]: nd, reaction time: 3hr, temp: RT | nd | Yes | >99% | 0.03 | nd | 23 |
| Annexin V | Isolink^a^ - (1mL; 2.5GBq) | Activity added: 1GBq, final volume: nd, [Final protein]: nd, reaction time: 60min, temp: 37°C, extra: blow dry rxn with N2 to reduce volume | >90% | Yes | >98% | nd | nd | 24 |
| *α- P185^HER-2^ scFv* | Homemade kit^b^ | Activity added: nd, final volume: nd, [Final protein]: 0.1mg/mL, reaction time: 60min, temp: 37°C, | <70% | Yes | nd | nd | nd | 25 |
| UA20 scFv | Isolink^a^ - (1mL; < 740MBq) | Activity added: 308MBq, final volume: 0.5mL, final protein Concentration: 0.18mg/mL, reaction time: 60min, temp: 37°C, | 55-65% | Yes | >95% | 7.4 | nd | 26 |
| M12 scFv | (3mL up to 30GBq); [^99m^Tc(CO)_3_]^+^ RCP >90% | Activity added: nd, final volume: 100mL, final protein Concentration: 1mg/mL, reaction time: 30min, temp: 37°C, | 70-95% | Yes | nd | 3.3 | nd | 1 |

^a^ Kit prepared according to manufacturer’s instructions; ^b^ Kit prepared according to Waibel et al.; ^c^ Kit prepared according to Alberto et al.; ^d^ Kit prepared according to Stalteri et al.; ^e^ nd = not described. LE = labelling efficiency, RCP = radiochemical purity, SA = specific activity,

**Effect of protein concentration, pH, and temperature on [^99m^Tc(CO)_3_]^+^** **radiolabelling of a His-tagged protein**

Recombinant CR2 (27), a 16 kDa His-tagged protein, was radiolabelled using IsoLink kit according to manufacturer’s instructions. General labelling was 1 mg/mL protein (62.5 µM), pH 7, 37 ^o^C, and 500 MBq [^99m^Tc(CO)_3_]^+^. The pH of the [^99m^Tc(CO)_3_]^+^::rCR2 reaction mixture was adjusted and compared at pH of 5, 7, and 9. Additionally, total protein concentration in the reaction mixture was adjusted and compared at 0.5 mg/mL (31.25 µM), 1 mg/ml (62.5 µM), and 2 mg/ml (125 µM). Finally the incubation temperature of the reaction mixture was adjusted and compared at RT, 37 ^o^C, and 50 ^o^C (Fig. S1). Radiolabelling at a low pH of 5 significantly slowed the reaction rate, and maximum SA and LE was not reached within the 2 h incubation. Labelling at the higher pH achieved maximum SA and LE within 30 min, with no significant improvement when increasing pH from 7 to 9 (Fig. S1a). Radiolabelling at RT, maximum SA and LE was reached within 2 h incubation. Labelling at the higher temperatures of 37 ^o^C and 50 ^o^C, increased the rate maximum SA and LE were reached >4 fold (Fig. S1b). Radiolabelling at a low protein concentration of 0.5 mg/mL significantly slowed the reaction rate, and maximum SA and LE was not reached within the 2 h incubation. Increasing the concentration to 1 mg/mL and 2 mg/mL, maximum SA and LE was reached within 1 h and 30 min respectively (Fig. S1c).

***Fig S1:*** *Effect of (a) pH, (b) temperature, and (c) protein concentration on [^99m^Tc(CO)_3_]^+^ radiolabelling of His-tagged protein rCR2 (n=3). Recombinant CR2 was radiolabelled with ~500 MBq [^99m^Tc(CO)_3_]^+^ while varying reaction conditions. Radiolabelling efficiencies were determined using iTLC over at 5, 15, 30, 60, 90, and 120 min. Error bars represent the standard deviation of the mean of the triplicate.*

**Calculation of total Na^+^ concentration when radiolabelling His-tagged proteins with [^99m^Tc(CO)_3_]^+^**

Salt concentrations were calculated based on the total [Na^+^] in the reaction mixture. This includes Na^+^ in the IsoLink Kit, Na^+^ in the [^99m^TcO_4_]^–^ saline eluent, Na^+^ in the protein solution, and added NaCl to make up the final salt concentration. An example calculation is illustrated below for the α-PSMA scFv protein used in this study:

*Salt in IsoLink Kit:*

| **Compounds** | **Mass (mg)** | **MW (g/mol)** | **Moles (mol)** | **Moles of Na^+^ (mol)** | **Stoichiometry** |
| --- | --- | --- | --- | --- | --- |
| Na2H3BCO2 | 4.5 | 103.82 | 4.33442E-05 | 8.66885E-05 | 2 |
| Na2B4O7.2H2O | 2.85 | 381.37 | 7.47306E-06 | 1.49461E-05 | 2 |
| Na2C4H4O6.2H2O | 8.5 | 230.08 | 3.69437E-05 | 7.38873E-05 | 2 |
| Na2CO3 | 7.15 | 105.99 | 6.74592E-05 | 0.000134918 | 2 |
|  | **Total moles of Na^+^ in 1mL IsoLink kit: 0.00031044** | | | |  |

*Salt in [^99m^TcO_4_]^–^ saline eluent:*

The concentration of Na^+^ in the IsoLink kit is very high and its contribution to the protein labelling will depend on the volume of [^99m^TcO_4_]^–^ in saline added to the kit as well as the amount of 1 M HCl added to neutralise it once the [^99m^Tc(CO)_3_]^+^ has been produced.

**Total kit volume =** Volume of [^99m^TcO_4_]^–^ in saline eluent + Volume of 1M HCl = 400 µL + 160 µL = 560 µL

**Volume of kit used** = Volume of the [^99m^Tc(CO)_3_]^+^ added to α-PSMA scFv is 10 µL. This can be used to calculate the moles of Na^+^ added and consequently the concentration.

|  | **Moles of Na^+^ from kit** | **Moles of Na^+^ from saline** | **Total moles of Na^+^ in ^99m^Tc kit** | **Total Volume of kit (µL)** | **Volume of kit used (µL)** | **Moles of Na^+^ used** |
| --- | --- | --- | --- | --- | --- | --- |
| **Low salt 0.25M** | 0.00031044 | 6.2069E-05 | 0.000372509 | 560 | 10 | 6.65195E-06 |

*Salt in protein solution:*

The concentration of [Na^+^] in the protein solution also needs to be considered. α-PSMA scFv was in PBS with a standard 0.14 M [Na^+^].

|  | **Moles of Na^+^ from kit** | **Volume of kit added to protein (µL)** | **[Na^+^] (Protein) (M)** | **Volume of Protein (µL)** | **Moles of Na^+^ (protein)** | **Total volume (protein + kit)** | **Total moles (protein + kit)** | **Total [Na^+^] (protein + kit)** |
| --- | --- | --- | --- | --- | --- | --- | --- | --- |
| **Low salt 0.25M** | 6.65E-06 | 10 | 0.14 | 20 | 0.0000028 | 30 | 9.45E-06 | 0.32M |

*Final salt concentration:*

In this study NaCl was used to adjust the final [Na^+^] in the labelling reactions to 0.25 M, 0.44 M and 0.63 M. The volume of the additional NaCl solution must also be accredited for:

|  | **Volume (protein & kit)** | **Moles Na^+^ (protein & kit)** | **Conc. of Na^+^ (NaCl soln.)** | **Vol. of NaCl soln.** | **Moles of Na^+^ (0.6M soln.)** | **Total Moles of Na^+^**  **(reaction mixture)** | **Total Volume**  **(reaction mixture)** | **Total [Na+] (reaction mixture)** |
| --- | --- | --- | --- | --- | --- | --- | --- | --- |
| **Low salt 0.25M** | 30 | 9.45E-06 | 0.05 | 10 | **0.0000005** | 9.95195E-06 | 40 | **0.25** |
| **Mid salt 0.44M** | 30 | 9.45E-06 | 0.8 | 10 | **0.000008** | 1.7452E-05 | 40 | **0.44** |
| **High salt 0.63M** | 30 | 9.45E-06 | 1.6 | 10 | **0.000016** | 2.5452E-05 | 40 | **0.63** |
